# Effect of Oligogalacturonides on Seed Germination and Disease Resistance of Sugar Beet Seedling and Root

**DOI:** 10.3390/jof8070716

**Published:** 2022-07-08

**Authors:** Can Zhao, Chunyan Wu, Kuikui Li, John F. Kennedy, Michael Wisniewski, Lihong Gao, Chenggui Han, Jia Liu, Heng Yin, Xuehong Wu

**Affiliations:** 1 College of Plant Protection, China Agricultural University, Beijing 100193, China; zhaocan19900809@163.com (C.Z.); wing201424@163.com (C.W.); hanchenggui@cau.edu.cn (C.H.); 2 College of Horticulture, China Agricultural University, Beijing 100193, China; gaolh@cau.edu.cn; 3 Dalian Engineering Research Center for Carbohydrate Agricultural Preparations, Liaoning Provincial Key Laboratory of Carbohydrates, Dalian Institute of Chemical Physics, Chinese Academy of Sciences, Dalian 116023, China; lkk@dicp.ac.cn; 4 Chembiotech Laboratories, Kyrewood House Tenbury Wells, Worcestershire WR15 8SG, UK; jfk@chembiotech.co.uk; 5 Department of Biological Sciences, Virginia Polytechnic Institute and State University, 220 Ag Quad Ln, Blacksburg, VA 24061, USA; wvwisniewski@gmail.com; 6 Chongqing Key Laboratory of Economic Plant Biotechnology, College of Landscape Architecture and Life Science/Institute of Special Plants, Chongqing University of Arts and Sciences, Chongqing 402160, China; liu.jia1983@hotmail.com

**Keywords:** antioxidant response, oligogalactouronide preparation, damage-associated molecular patterns, seedling damping-off, root and crown rot, sugar beet

## Abstract

Oligogalacturonides (OGs) are a bioactive carbohydrate derived from homogalacturonan. The OGs synthesized in this study significantly inhibited the mycelial growth of *Rhizoctonia solani* AG-4HGI in vitro, even at a low concentration (10 mg/L). The seed vigor test demonstrated that the application of 50 mg/L OGs to sugar beet seeds significantly increased average germination percentage, germination energy, germination index, and seedling vigor index. The same concentration of OGs also improved the seedling emergence percentage of sugar beet when seeds were sown in soil inoculated with D2 and D31 isolates, respectively. The lesion diameter on mature sugar beet roots caused by *R. solani* AG-4HGI isolates D2 and D31 also decreased by 40.60% and 39.86%, respectively, in sugar beets roots first treated with 50 mg/mL OGs in the wound site, relative to lesion size in untreated/pathogen inoculated wounds. Sugar beet roots treated with 50 mg/mL OGs prior to inoculation with the D2 isolate exhibited up-regulation of the defense-related genes glutathione peroxidase (GPX) and superoxide dismutase (SOD) by 2.4- and 1.6-fold, respectively, relative to control roots. Sugar beet roots treated with 50 mg/mL OGs prior to inoculation with D31 exhibited a 2.0- and 1.6-fold up-regulation of GPX and SOD, respectively, relative to the control. Our results indicate that OGs have the potential to be used for the protection of sugar beet against *R. solani* AG-4HGI.

## 1. Introduction

Oligogalacturonides (OGs) are oligomers of 1,4-linked alpha-D-galacturonosyl residues, a major component of pectin, that are produced by the degradation of homogalacturonan [1,2,3]. OGs have been reported to affect plant metabolism in a variety of ways, including eliciting a wide range of defense responses that confer protection against plant pathogens [3,4,5], regulating plant growth and development [6,7], and acting as signaling molecules in plants by activating a Ca^2+^-mediated signaling pathway [5,8]. The defense responses elicited by OGs include the accumulation of phytoalexins [9,10], glucanases, and chitinases [11], activation of salicylic acid and jasmonic acid hormone pathways [12], deposition of callose [13], production of reactive oxygen species [12,14,15,16], and generation of nitric oxide [12,17,18]. The bioactive characteristics of OGs are influenced by their structure and degree of polymerization (DP). In this regard, the induction of plant defense response by OGs occurs in response to the use of OGs with a high DP (from 10 to 15) [3,19]. Shorter oligomers, however, have also been recently reported to activate some defense responses in a manner similar to those described for long OGs [3,20].

Sugar beet (*Beta vulgaris* L.) is the second-largest sugar crop produced worldwide. *Rhizoctonia* is a major pathogen of sugar beet that infects plants throughout the entire cultivation cycle, causing sugar beet seedling damping-off, as well as root and crown rot, which result in severe yield losses and a decrease in the level of saccharinity [21]. Among the anastomosis groups (AGs) or subgroups of *Rhizoctonia*, isolates of *R. solani* AG-4HGI are the principle causal agents of seedling damping-off, as well as root and crown rot in sugar beet [22,23,24,25,26]. OGs are now considered to function as damage-associated molecular patterns (DAMP) that activate plant innate immunity [3,27,28]. Therefore, we hypothesized that OGs applied to sugar beet seeds could protect plants against *R. solani* AG-4HGI throughout the entire cultivation cycle.

The objective of the current study was to prepare OGs and determine their effect on sugar beet seed vigor, including the germination percentage, germination energy, germination index, and seedling vigor index. We also wanted to evaluate the ability of the OGs to inhibit the growth of *R. solani* AG-4HGI in vitro, and their ability to control sugar beet seedling damping-off and root rot. The ability of OGs to induce defense-related gene expression in sugar beets, including glutathione peroxidase (*GPX*) and superoxide dismutase (*SOD*), was also determined.

## 2. Materials and Methods

### 2.1. Preparation and Characterization of OGs

The preparation of oligogalacturonides (OGs) was achieved through endo-polygalacturonase (*An*PG28A) [29] degradation of sunflower plate pectin whose molecular weight, degree of esterification, and uronic acid content are about 280 kD, 24%, and 90%, respectively. *An*PG28A acts on the alpha-1,4-linked galacturonic acid backbone in pectin through endo-mode. The reaction was carried out in a water-based solution containing 10% (*w*/*v*) sunflower plate pectin and 50 μmol *An*PG28A at 30 °C for 36 h. The final products were obtained by spray drying after the removal of insoluble matter by centrifugation. The DPs of the OGs were determined by Electrospray-ionization mass spectra (ESI-MS) obtained with an Agilent 6540 UHD Q-TOF system (Santa Clara, CA, USA) in the negative ion mode. The resulting data were analyzed using MassHunter Workstation Data Acquisition (Santa Clara, CA, USA) software-Qualitative Analysis B.06.00.

### 2.2. Plant Materials

Sugar beet (*B. vulgaris* cv. NTG9903) seeds with single embryos were used in this study. Sugar beet seeds were pretreated by washing under running tap water for 4 h to remove natural compounds that are present in the seed coat that inhibit seed germination. They were then dried at 25 °C. The prepared sugar beet seeds were then surface disinfected by soaking in 1% sodium hypochlorite for 5 min, followed by five rinses in sterilized water, after which they were placed on sterile filter paper on a clean laboratory bench for air drying.

Treatments were administered by soaking the air-dried seeds in a 50 mg/L solution of OGs for 30 min. Control seeds were soaked in an equal volume of sterile distilled water for 30 min. Additional plant materials consisted of sugar beet roots harvested at commercial maturity that were of uniform size (average fresh weight of 800 g) and without any evidence of rot. All the roots were first washed under running tap water and then surface disinfected by soaking in 1% sodium hypochlorite for 5 min, followed by five rinses in sterilized water and air drying on a clean, laboratory bench.

### 2.3. Fungal Pathogens

Two isolates of *R. solani* AG-4HGI (D2 and D31) were obtained from diseased sugar beets collected in Chifeng city, Inner Mongolia autonomous region, and Qiqihar city, Heilongjiang province, China, respectively. The two isolates were identified based on their morphological characteristics and sequence analysis of the internal transcribed spacer region of ribosomal DNA (rDNA-ITS). The isolates were maintained on sterile barley grain at 4 °C [26] and reactivated on potato dextrose agar (PDA) plates prior to use.

### 2.4. The Effect of OGs on Mycelial Growth of R. solani AG-4HGI

The mycelial growth rate method was used to assess the effect of OGs on the mycelial growth of *R. solani* AG-4HGI isolates in vitro. OGs were first dissolved in sterilized distilled water at a concentration of 10, 50, 100, 200, and 400 g/L, and then appropriate volumes of each concentration of OGs were added to autoclaved PDA cultural media after sufficient cooling to approximately 45 °C but prior to solidification to obtain final concentrations of 10, 50, 100, 200, and 400 mg/L. Equivalent amounts of sterilized distilled water were added to non-amended PDA culture media to serve as a control. Mycelial plugs (5 mm in diameter) were cut from the margin of actively growing colonies of three-day-old cultures of *R. solani* AG-4HGI isolates (D2 and D31). One plug was placed in the center of OGs-amended and unamended PDA plates with three replicate plates for each isolate-OGs combination. Colony diameters were measured at two perpendicular axes after plates were incubated for 48 h at 25 °C in the dark. The experiment was repeated three times.

### 2.5. Assessment of OGs Effect on the Germination of Sugar Beet Seeds

A sugar beet seed germination assay was conducted using three bio-replicates of 100 seeds in each replicate, thus, assessing the effect of each treatment on the viability of 300 seeds. The assay was used to determine the effect of two treatments: seeds treated with 50 mg/L OGs, and control samples using untreated seeds. One hundred seeds were placed on three-layered filter paper moistened with 20 mL of sterile distilled water in a germination box, and then covered with another sheet of three-layered moistened filter paper. The germination box was then placed in a growth chamber in the dark at 25 °C with the relative humidity maintained at >95%. Sugar beet seeds were considered germinated when a radicle of at least 2 mm emerged. Seed germination was recorded daily for 14 days as described by ISTA [30].

Several germination indices were also assessed to evaluate the effect of the OGs treatments, including germination percentage (GP), germination energy (GE), germination index (GI), and seedling vigor index (SVI). These parameters were calculated as described by Puglisi et al. [31] and detailed below:

Germination percentage = (number of germinated seeds/number of total seeds for bioassay) × 100;

Germination energy *=* (number of germinated seeds after germination for 4 days/number of total seeds sets for bioassay) × 100;

Germination index = [number of germinated seeds/days of first count] + … + [number of germinated seeds/days of final count];

Seedling vigor index = GI × average seedling length on the day of the final count.

### 2.6. Ability of OGs to Prevent Sugar Beet Seedling Damping-Off Caused by R. solani AG-4HGI

The ability of OGs to prevent sugar beet seedling damping-off caused by *R. solani* AG-4HGI was assessed in the greenhouse conditions using the method of Bolton et al. [32]. The soil used was a mixture of sand and sawdust (1:2 *v*/*v*, dry heat sterilization at 161 °C for 4 h before use) placed in a plastic pot (top diameter × bottom diameter × height: 90 mm × 70 mm × 90 mm). The inoculum was ground *Rhizoctonia*-infested barley mixed with the sterilized soil mixture at a final rate of 0.4 CFU (colony forming units)/g of soil.

To prepare the inoculum, the *R. solani* AG-4HGI (D2 and D31) isolates were allowed to grow on PDA plates for 7 days and then transferred to a sterile barley medium (100 cm^3^ of barley seeds soaked in 60 mL of distilled water and autoclaved at 121 °C for 75 min). The infested barley was removed from the flasks 2 weeks after it was inoculated with *R. solani* AG-4HGI, air-dried for 2 days, and then ground in a blender. To ensure that the inoculum load was similar among the tested isolates, three 10 mg samples of ground barley inoculum from each isolate were placed on individual plates of PDA and incubated at 25 °C. The number of CFU was counted after 18 h.

Each plastic pot was filled with 250 g soil mix containing *R. solani* AG-4HGI isolates with 0.4 CFU/g of soil. Then 20 seeds pre-treated with OGs or 20 untreated sugar beet seeds were planted in each separate pot. The experimental design was a randomized complete block with three replicates (one pot per replicate) of each treatment per isolate. All the plants were incubated under greenhouse conditions that were maintained at 25 to 27 °C with a 12 h photoperiod. The number of surviving seedlings was recorded after 3 weeks. The experiment was conducted twice.

Seedling emergence (%) was calculated as follows: seedling emergence = (number of seedlings surviving 3 weeks after planting in soil inoculated with *R. solani* AG-4HGI/number of total seeds that sowed) × 100.

### 2.7. Ability of OGs to Prevent Sugar Beet Root Rot Caused by R. solani AG-4HGI

The ability of OGs to control sugar beet root rot caused by *R. solani* AG-4HGI was evaluated using the method previously described by Liu et al. [33] and Wang et al. [34] with a slight modification. Harvested mature sugar beet roots were wounded (3 mm deep × 3 mm wide) at the equator using a sterile nail. Then, 10 μL of a 50 mg/L solution of OGs or sterile distilled water (control) was pipetted into each wound. The wound was air-dried on a clean laboratory bench for 2 h and then a mycelial disk of one of the *R. solani* AG-4HGI isolates was applied to each wound. Treated sugar beet roots were placed on Petri dishes in covered plastic boxes (190 mm × 140 mm × 50 mm) with sterile water to maintain a high relative humidity (about 95%) and stored at 25 °C. Lesion diameter in the sugar beet roots caused by *R. solani* AG-4HGI was measured after 14 days. Each treatment comprised of three biological replicates, and each biological replicate comprised of 10 sugar beet roots. The assay was conducted three times. Tissue samples (approximately 100 mg) around the wound site, consisting of both skin and flesh, were obtained at the end of the experiment (14 days) from 30 roots under aseptic conditions and pooled for the analysis of gene expression.

### 2.8. Effect of OGs on the Expression of Defense-Related Genes in Sugar Beet Roots

Approximately 100 mg of sugar beet tissue from each sample was used to extract total RNA, using an RNAprep Pure Plant Plus Kit (Polysaccharides & Polyphenolics-rich) (Tiangen, Beijing, China) according to the manufacturer’s protocol. The extracted RNA was then treated with DNase I to remove any genomic DNA. RNA concentration was measured using UV spectrophotometry (Nanodrop 2000, Thermo, Waltham, MA, USA), and RNA integrity was evaluated on a 1% agarose gel. First-strand cDNA synthesis was performed using a High-Capacity cDNA Reverse Transcription Kit with RNase Inhibitor (TaKaRa, Dalian, China). Reverse transcription-quantitative PCR (RT-qPCR) analysis was performed using SYBR Green PCR Master Mix (TaKaRa) according to the manufacturer’s protocol. The RT-qPCR analysis was conducted in 96-well plates with three biological replicates for each treatment/sample combination, and the assessment was conducted three times. Results were analyzed using ABI QuantStudioTM 6 Flex System (Applied Biosystems, Foster City, CA, USA) software. The expression of glyceraldehyde-3-phosphate dehydrogenase (*GAPDH*) was used as a housekeeping gene. Relative gene expression was calculated using the 2^−ΔΔCt^ method and normalized to the level of the samples treated without OGs in steady-state. RNA from the samples that were not treated with OGs was used as a calibrator. The gene-specific primers used in the RT-qPCR analysis are listed in Table 1.

### 2.9. Data Analysis

All statistical analyses were performed using SPSS version 20.0 software (SPSS Inc., Chicago, IL, USA). Data with a single variable (treatment) were analyzed using a one-way analysis of variance (ANOVA), and mean separations were performed using Duncan’s multiple range test or a Student’s *t*-test. Statistical analysis of data obtained from the non-OGs control and 50 mg/L OGs treatment were utilized a Student’s *t*-test. Differences at *p* < 0.05 were considered as significant. Data obtained from three independent repeated experiments were pooled, as the interaction between treatment and experiment was determined to be not significant.

## 3. Results and Discussions

### 3.1. Preparation and Characterization of OGs

**Figure 1:**
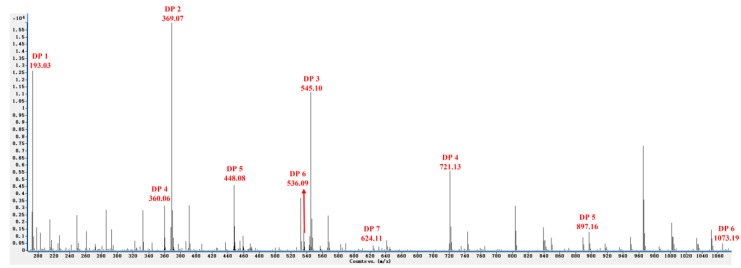
ESI-MS analysis in the negative ion mode revealing different degrees of polymerization of oligogalacturonides (OGs).

The composition of the reaction products was analyzed by ESI-MS in the negative ion mode. Results demonstrated that a series of oligosaccharides with different DPs (DP 2–7, the average molecular weight of them is about 770 Da, and contain esterification groups) were created through the enzymatic degradation of sunflower plant pectin (Figure 1). The mass-to-charge ratios (*m*/*z*) of OGs in the negative ion mode were calculated as DP 2 (369.07 *m*/*z*), DP 3 (545.10 *m*/*z*), DP 4 (721.13 *m*/*z*, double-charged fragment: 360.06 *m*/*z*), DP 5 (897.16 *m*/*z*, double-charged fragment: 448.08 *m*/*z*), DP 6 (1073.19 *m*/*z*, double-charged fragment: 536.09 *m*/*z*), and DP 7 (double-charged fragment: 624.11 *m*/*z*). The degree of polymerization (DP) and the structure of oligosaccharide are closely related to their biological activity. Previous studies reported that OGs with a high DP (from 10 to 15) could effectively induce a strong plant defense response [3,19], however, shorter oligomers have also been recently reported to activate defense responses [3,20] in a manner similar to long OGs. We hypothesized that the OGs prepared using the methodology described in the present study would induce a disease defense response in sugar beets that would enhance their resistance to *R. solani* AG-4HGI. Therefore, further studies were conducted.

### 3.2. Inhibitory Effect of OGs on Mycelial Growth of R. solani AG-4HGI

**Figure 2:**
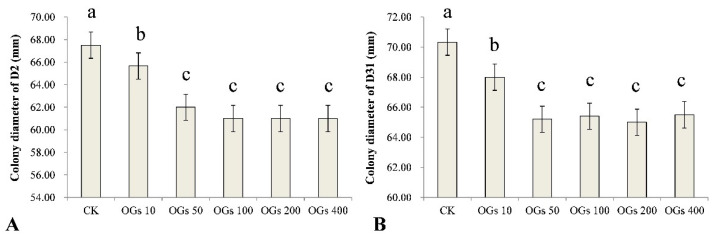
Effect of different concentrations of oligogalacturonides (OGs) on the mycelial growth of *R. solani* AG-4HGI in vitro. (**A**) Colony diameter of the D2 isolate of *R. solani* AG-4HGI grown on PDA amended with different concentrations of OGs. (**B**) Colony diameter of the D31 isolate of *R. solani* AG-4HGI grown on PDA amended with different concentrations of OGs. CK: *R. solani* AG-4HGI isolates grown on non-amended PDA plates; OGs 10, 50, 100, 200, and 400 mg/L: *R. solani* AG-4HGI isolates grown on PDA plates amended with OGs at concentrations of 10, 50, 100, 200, and 400 mg/L, respectively. Columns in each graph with different letters indicate significantly different means according to a Duncan’s multiple range test at *p* < 0.05.

Mycelial growth is essential for a pathogen to be able to infect a plant host and inhibition of that growth may represent a significant component of the antifungal activity of a compound. In our study, we evaluated five concentrations (10, 50, 100, 200, and 400 mg/L) of OGs, using sterilized distilled water as a control, to determine the effect of the synthesized OGs on the mycelial growth of *R. solani* AG-4HGI in vitro. We determined whether the level of inhibitory activity, if present, was concentration-dependent. Results indicated that the OGs significantly inhibited the mycelial growth of the D2 and D31 isolates of *R. solani* AG-4HGI even at a low concentration (10 mg/L) (Figure 2 and Figure S1). Notably, however, the inhibitory effect did not increase incrementally with the use of increasing concentrations of OGs, and no significant differences in activity were observed in the range of 50–400 mg/L (Figure 2).

Other studies have also reported similar results and noted that lower concentrations of different oligosaccharides have a stronger effect on stimulating plant growth and enhancing host resistance to various pathogens. Howlader et al. [12] reported that the effect of OGs on the induction of disease resistance in *Arabidopsis* decreased as OGs concentration increased from 25–200 mg/L. Davidsson et al. [20] also demonstrated that higher concentrations (up to 10 mM) of OGs did not induce an oxidative burst of reactive oxygen species (ROS). It has been suggested that the receptor for OGs has a specific concentration requirement for recognition and that high concentrations of oligosaccharides may simply oversaturate the receptors, as well as cause some other side effects [35].

### 3.3. Effect of OGs on Sugar Beet Seed Germination

**Figure 3:**
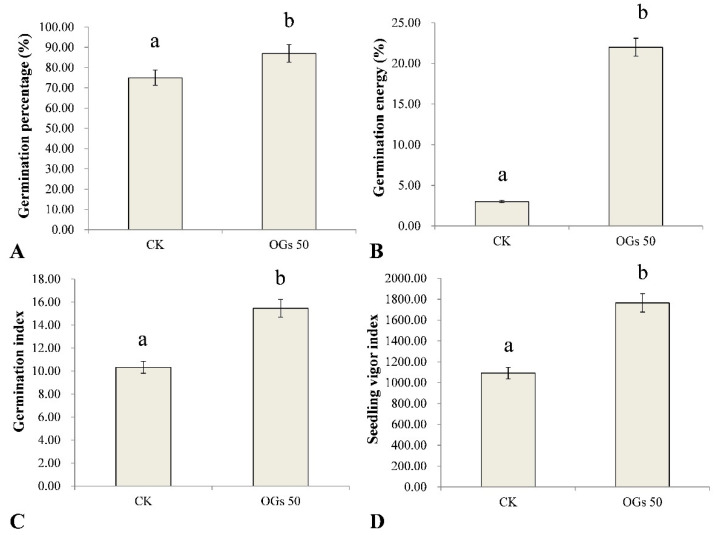
Effect of 50 mg/mL oligogalacturonides (OGs) on sugar beet seed germination. (**A**) Effect of OGs on the average germination percentage of sugar beet seeds. (**B**) Effect of OGs on the average germination energy of sugar beet seeds. (**C**) Effect of OGs on the average germination index of sugar beet seeds. (**D**) Effect of OGs on the average seedling vigor index of sugar beet seeds. CK: Untreated sugar beet seeds; OGs 50: Sugar beet seeds treated with 50 mg/L of OGs. Data represent the mean ± standard deviation of three independent experiments where each experiment consisted of three biological replicates (*n* = 100). Columns in each graph with different letters indicate significantly different means according to Duncan’s multiple range test at *p* < 0.05.

Seed vigor is the sum of the properties determining the ability of seed lots to exhibit acceptable levels of germination in a wide range of environments. In this study, the effect of OGs on several germination indices in sugar beet seeds was evaluated (Figure S2). Germination percentage, germination energy, germination index, and the seedling vigor index of sugar beet seeds were significantly enhanced by treating seeds with 50 mg/L OGs (Figure 3). The average germination percentage of sugar beet seeds treated with 50 mg/L OGs was 87%, which was significantly higher than the level of germination of untreated seeds (75%) (Figure 3A). Similarly, the germination energy of sugar beet seeds treated with 50 mg/L OGs (22%) was approximately 7.3 times higher than it was in untreated seeds (3%) (Figure 3B). In addition, the germination index and seedling vigor index of sugar beet seeds were also significantly enhanced approximately 1.5 and 1.6 times, respectively, when seeds were treated with 50 mg/L OGs (Figure 3C,D). The enhancement of seed germination by treatment of seeds with oligosaccharides has been previously reported. Hu et al. [36] reported that alginate-derived oligosaccharides enhanced the rate of maize seed germination by increasing the activity of α- and β-amylase. In addition, Udchumpisai et al. [37] reported that pectic-oligosaccharides treatments considerably decreased the germination time of rice seeds by 17–19%. They alsoincreased the seedling root and shoot length as well as the amylase activity of seedlings at 5 days after sowing compared with the control (rice seeds not treated with pectic-oligosaccharides).

Seeds with high germination energy typically also have higher seedling vigor and dry matter weight, as well as enhanced stress tolerance to both abiotic and biotic stresses, including fungal pathogens [38]. The germination index is an indicator of seed vigor under stress conditions, and a higher germination index indicates a higher number of vigorous seeds. In this regard, a pre-sowing treatment of seeds with different bioactive compounds was reported to increase the vigor of seedlings and enhance their ability to tolerate different kinds of stresses [39]. Therefore, our results indicate that the use of OGs as a priming treatment of sugar beet seeds may represent a potential management practice to enhance seed germination and seedling vigor.

### 3.4. Ability of OGs to Prevent Sugar Beet Damping-Off and Root Rot Caused by R. solani AG-4HGI

**Figure 4:**
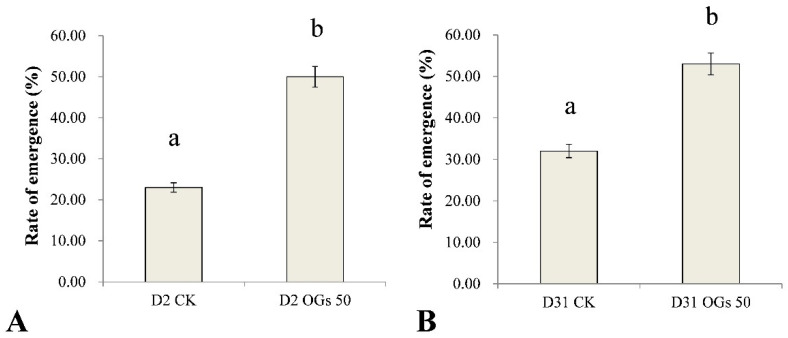
Ability of oligogalacturonides (OGs) to prevent sugar beet seedling damping-off caused by *R. solani*. (**A**) Rate of sugar beet seedling emergence in soil inoculated with *R. solani* AG-4HGI isolate D2. (**B**) Rate of sugar beet seedling emergence in soils inoculated with *R. solani* AG-4HGI isolate D31. CK: Emergence of non-treated sugar beet seeds; OGs 50: Emergence of sugar beet seeds treated with 50 mg/L OGs. Data represent the mean ± standard deviation of three independent experiments where each experiment consisted of three biological replicates (*n* = 20). Columns in each graph with different letters indicate significantly different means according to Duncan’s multiple range test at *p* < 0.05.

*Rhizoctonia solani* AG-4HGI is the predominant pathogen associated with sugar beet seedling damping-off and root and crown rot [24,26]. In the present study, seedling emergence rate after sowing seeds in soil inoculated with *Rhizoctonia* was used to evaluate the virulence of *Rhizoctonia* isolates on sugar beet seedlings, while lesion diameter was used as an indication of the severity of sugar beet root rot. The emergence percentage of untreated sugar beet seedlings was 23% and 32% when seeds were sown in soil inoculated with D2 and D31 isolates of *R. solani* AG-4HGI, respectively (Figure 4). In contrast, when seeds were treated with 50 mg/mL OGs, the percentage of seedling emergence in D2 and D31 inoculated soils was 50% and 53%, respectively (Figure 4). The results indicate that OGs at a concentration of 50 mg/mL could enhance resistance to *Rhizoctonia*, which improved seedling emergence by reducing the incidence of seedling damping-off.

**Figure 5:**
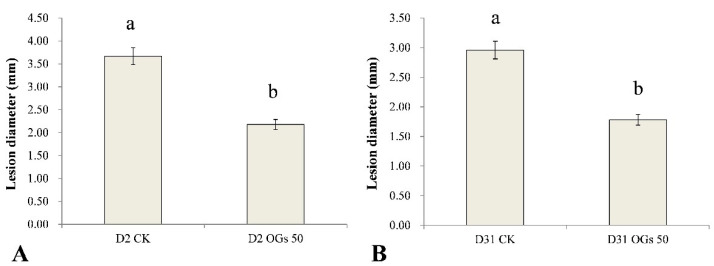
Ability of oligogalacturonides (OGs) to prevent mature sugar beet root rot caused by *R. solani* AG-4HGI. (**A**) Lesion diameter in sugar beet roots inoculated with *R. solani* AG-4HGI isolate D2. (**B**) Lesion diameter in sugar beet roots inoculated with *R. solani* AG-4HGI isolate D31. CK: Untreated sugar beet roots; OGs 50: Sugar beet roots treated with 50 mg/L of OGs. Data represent the mean ± standard deviation of three independent experiments where each experiment consisted of three biological replicates (*n* = 10). Columns in each graph with different letters indicate significantly different means according to Duncan’s multiple range test at *p* < 0.05.

Similarly, the lesion diameter in the mature sugar beet roots resulting from the D2 and D31 isolates of *R. solani* AG-4HGI was 40.60% and 39.86% lower, respectively, in sugar beet roots first treated with 50 mg/mL OGs in the wound site, relative to lesion size in untreated/pathogen inoculated wounds (Figure S3 and Figure 5). These results indicate that OGs at a concentration of 50 mg/mL could enhance resistance to *Rhizoctonia* in mature sugar beet roots and thus reduce the incidence of root rot.

OGs have been reported to be one of several DAMPs capable of inducing a wide range of defense responses that confer protection against pathogens [3,4,5]. In a previous study, Randoux et al. [16] reported that wheat plants sprayed with OGs 48 h prior to inoculation with *Blumeria graminis* (DC.) E.O. Speer f. sp. *tritici* could reduce the rate of infection by 57–58%, relative to untreated controls. Additionally, the presence of naturally occurring OGs with a low degree of methylation in transgenic strawberry fruits overexpressing pectin methylesterases was correlated with a higher level of defense responses and partial resistance against *Botrytis cinerea* Pers.: Fr, relative to non-transgenic, strawberry plants [40]. Treatment of grapevine leaves with OGs also reduced infection of *B. cinerea* by 55–65% [41]. These studies provide examples of OGs acting as a bio-elicitor of disease resistance. In this regard, it was the first time to evaluate the ability of OGs to enhance the resistance of sugar beet seedlings and mature roots to the D2 and D31 isolates of *R. solani* AG-4HGI that are the causal agents of sugar beet seedling damping-off and root rot in the present study.

### 3.5. The Effect of OGs on Defense-Related Gene Expression in Sugar Beet Roots

**Figure 6:**
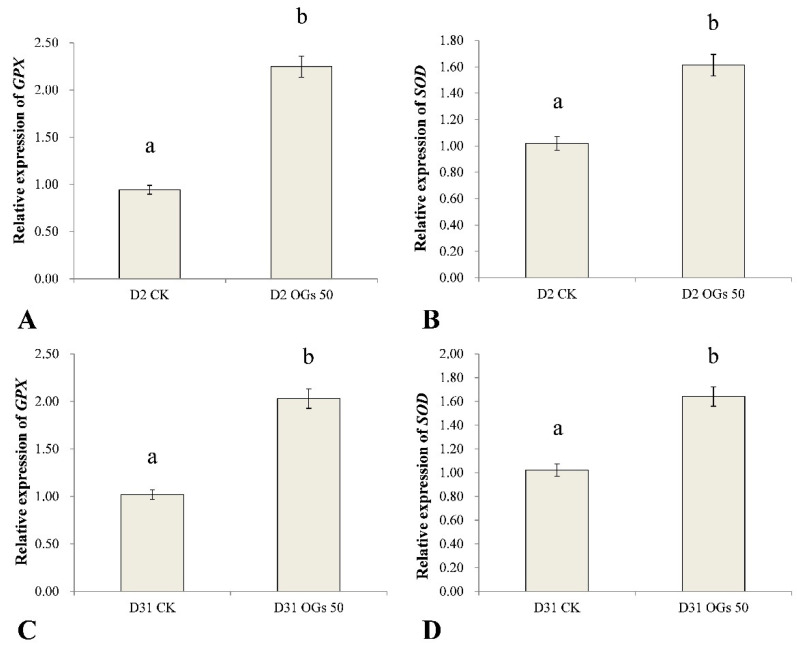
Effect of OGs on the relative expression of glutathione peroxidase (*GPX*) (**A**,**C**) and superoxide dismutase (*SOD*) (**B**,**D**) genes in harvested sugar beet roots. CK: Untreated sugar beet roots; OGs 50: Sugar beet roots treated with 50 mg/L of OGs. Data represent the mean ± standard deviation of three independent experiments where each experiment consisted of three biological replicates (*n* = 9). Columns with different letters in each graph indicate significantly different means between non-treated, control roots, and roots treated with 50 mg/L OGs according to a Student’s *t*-test at *p* < 0.05.

*GPX* and *SOD* are important antioxidant enzyme genes in plants that play important roles in eliminating ROS and ameliorating oxidative stress poisoning in plants. In general, antioxidants enhance abiotic and biotic stress tolerance in plants, including pathogen-induced stress. Therefore, in the present study, we used RT-qPCR to assess the relative level expression of *GPX* and *SOD* in sugar beet roots treated or not treated with OGs inoculated with *R. solani* AG-HGI isolates D2 and D31. Results indicated that *GPX* and *SOD* were significantly up-regulated in sugar beet roots treated with 50 mg/mL OGs, relative to untreated roots (Figure 6). *GPX* expression in sugar beet roots inoculated with the D2 isolate and treated with OGs was approximately 2.4-fold higher than it was in untreated roots (Figure 6A), and *SOD* expression was approximately 1.6-fold higher than it was in untreated roots (Figure 6B). *GPX* in sugar beet roots inoculated with the D31 isolate and treated with OGs was approximately 2.0 times higher than it was in untreated roots (Figure 6C), and *SOD* expression was approximately 1.6 times higher than it was in untreated roots (Figure 6D).

OGs can activate signaling pathways that induce rapid defense responses when a plant–pathogen interaction occurs. OGs trigger plant defense responses such as the production of ROS, production of anti-microbial metabolites, and the synthesis of pathogenesis-related proteins [5]. In the present study, we found that *GPX* and *SOD* expression, which contribute to the maintenance of ROS homeostasis and prevent oxidative injury, were both up-regulated. Similar results have been reported by Camejo et al. [42], who found that an increase in SOD activity in alfalfa roots treated with 50 and 100 mg/L OGs, and enhanced O^2-^ scavenging activity. Our results indicate that OGs can induce resistance to *R. solani* AG-4HGI in both seedlings and mature sugar beet roots.

## 4. Conclusions

The present study demonstrated that OGs in the range of 2–7 DPs (synthesized through the enzymatic degradation of sunflower plate pectin) acted as a bioactive elicitor in sugar beet seeds, seedlings, and harvested roots. The synthesized OGs significantly inhibited the mycelial growth of *R. solani* AG-4HGI in vitro at a concentration of 50 mg/L, increased the vigor of sugar beet seeds, reduced the occurrence of damping-off in sugar beet seedlings, reduced lesion diameter in harvested sugar beet roots inoculated with two different isolates (D2 and D31) of *R. solani* AG-4HGI, and enhanced the expression of defense-related genes (*GPX* and *SOD*) in harvested sugar beet roots. The findings of our study indicate that OGs represent a bioactive, natural compound that effectively enhances resistance to *R. solani* AG-4HGI in both seedlings and harvested sugar beet roots. Further studies utilizing OGs will be required to assess its potential commercial use on sugar beet.

## Figures and Tables

**Table 1:** Gene-specific primers used in the RT-qPCR analysis of gene expression.

Gene	GenBank Accession Number	Primer Sequence	Product Size (bp)
*GPX*	XM_019250362.1	F: AGCAGGCCGTGTCACTTTR: TATGCCCGACGACCAATC	193
*SOD*	XM_010672327	F: TGATTTGGCGTGGTTTCGR: ATCTTCACTGGGTTGGGCTAT	153
*GAPDH*	XM_010679634	F: GCTCAACATCGTCCCTCTR: TCTCAGCCTCACATTTCTC	118

## Data Availability

Data are contained within the article.

## Supplementary Materials

The following are available online at https://www.mdpi.com/article/10.3390/jof8070716/s1, Figure S1: Effect of oligogalacturonides (OGs) on the mycelial growth of *R**. solani* AG-4HGI in vitro. (A) Mycelial growth of *R. solani* AG-4HGI isolate D2 grown on potato dextrose agar (PDA) amended with 10, 50, 100, 200, and 400 mg/L of OGs and not amended with OGs (CK) for 48 h. (B) Mycelial growth of *R. solani* AG-4HGI isolate D31 grown on PDA amended with 10, 50, 100, 200, and 400 mg/L of OGs and not amended with OGs (CK) for 48 h. Figure S2: Effect of 50 mg/mL oligogalacturonides (OGs) on seed germination of sugar beet. (A) Sugar beet seedlings after germination from seeds not treated with OGs for 14 days; the three germination boxes are three replicates; (B) Sugar beet seedlings after germination from seeds treated with 50 mg/L OGs for 14 days; the three germination boxes are three replicates. Figure S3: Ability of oligogalacturonides (OGs) to prevent mature sugar beet root rot caused by *R. solani* AG-4HGI. (A) Lesion on sugar beet roots inoculated with *R. solani* AG-4HGI isolate D2, which were not treated with OGs. (B) Lesion on sugar beet roots inoculated with *R. solani* AG-4HGI isolate D2, which were treated with 50 mg/L OGs; (C) Lesion on sugar beet roots inoculated with *R. solan*i AG-4HGI isolate D31, which were not treated with OGs. (D) Lesion on sugar beet roots inoculated with *R. solani* AG-4HGI isolate D31, which were treated with 50 mg/L OGs.
