# Supplementary figures and images for: Effect of Oligogalacturonides on Seed Germination and Disease Resistance of Sugar Beet Seedling and Root
Source: J Fungi (Basel). 2022 Jul 8;8(7):716. doi: 10.3390/jof8070716 (PMC9323887; doi:10.3390/jof8070716)

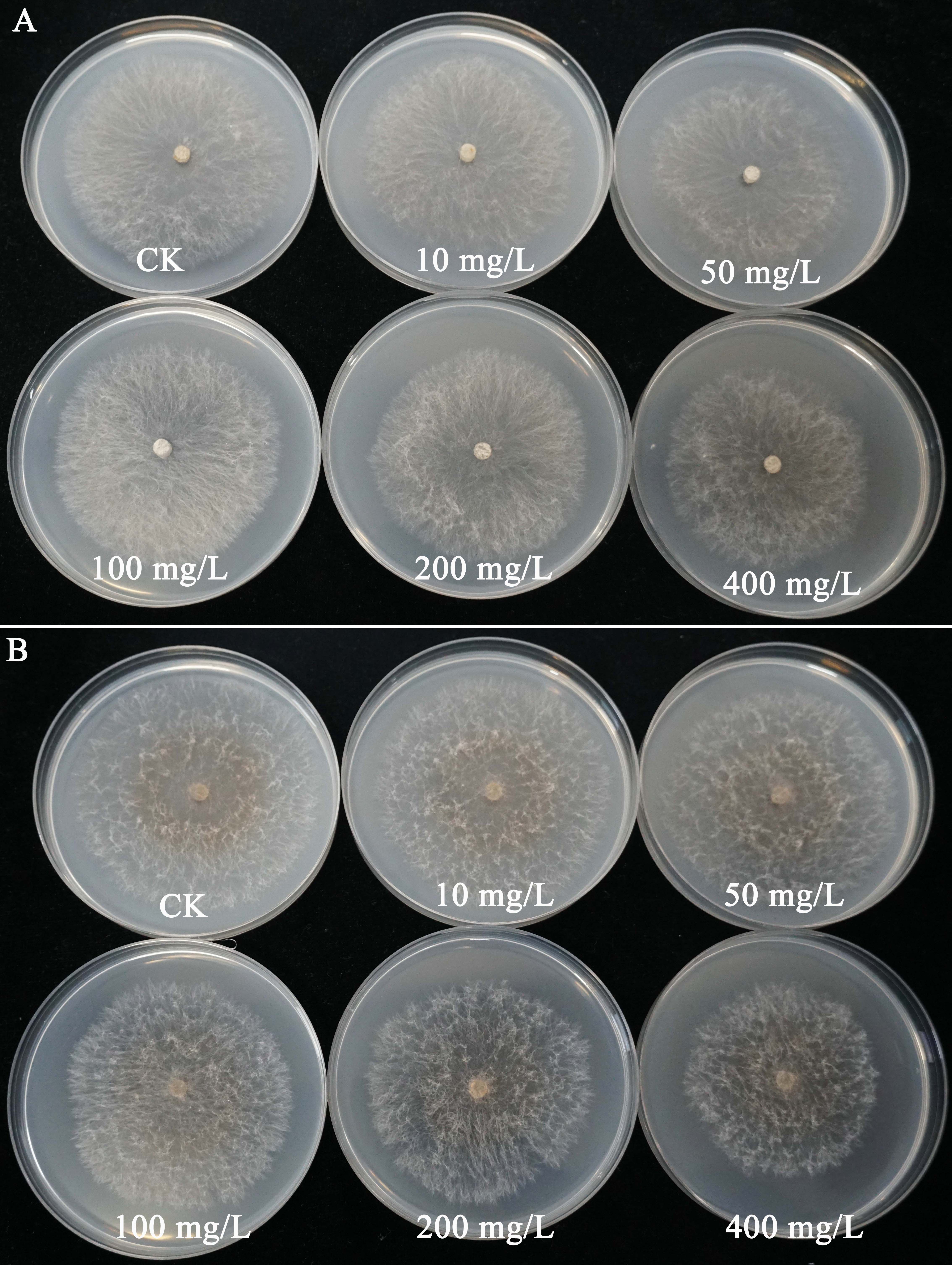

Supplement: Supplementary file 1 [file jof-08-00716-s001.zip › Figure S1.jpg]

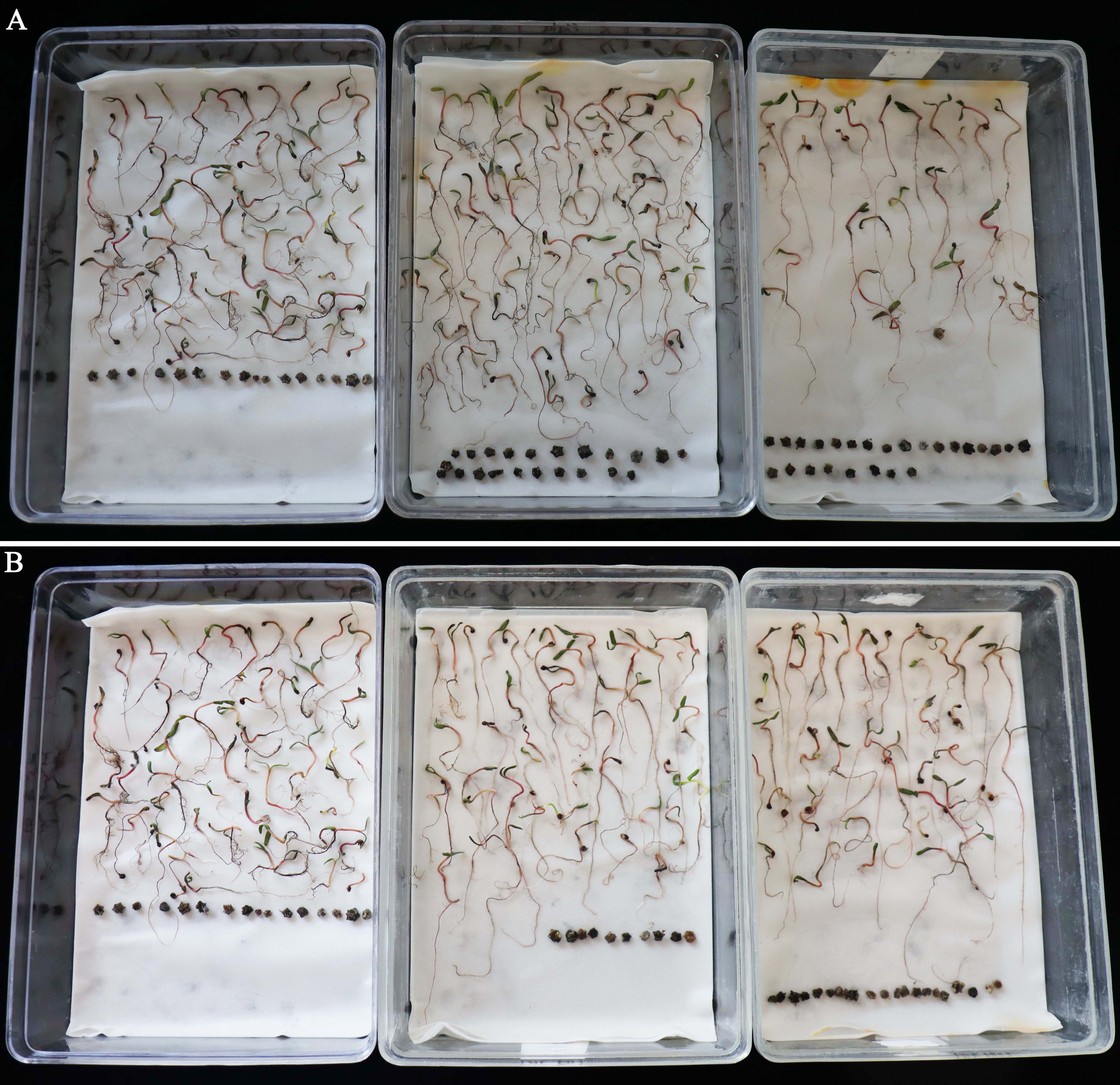

Supplement: Supplementary file 1 [file jof-08-00716-s001.zip › Figure S2.jpg]

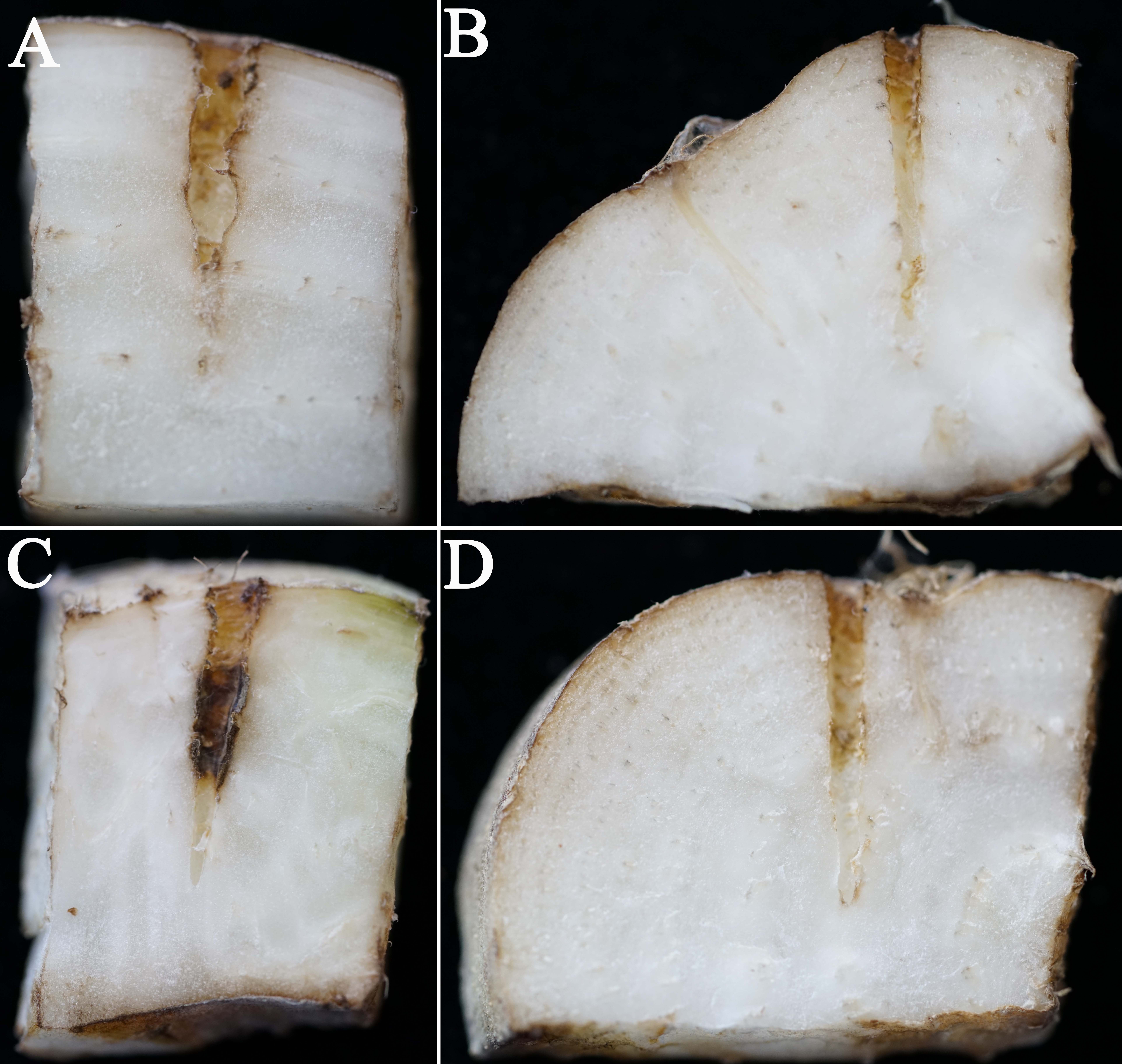

Supplement: Supplementary file 1 [file jof-08-00716-s001.zip › Figure S3.jpg]
